# Temporal Dynamics of Endogenous Bacterial Composition in Rice Seeds During Maturation and Storage, and Spatial Dynamics of the Bacteria During Seedling Growth

**DOI:** 10.3389/fmicb.2022.877781

**Published:** 2022-07-22

**Authors:** Swarnalee Dutta, Soo Yeon Choi, Yong Hoon Lee

**Affiliations:** ^1^Division of Biotechnology, Jeonbuk National University, Jeonju, South Korea; ^2^Crop Foundation Research Division, National Institute of Crop Science, Wanju-gun, South Korea; ^3^Plant Medical Research Center, Advanced Institute of Environment and Bioscience, Institute of Bio-Industry, Jeonbuk National University, Jeonju, South Korea

**Keywords:** endophyte, microbiome, PGPR, seed, temporal dynamics

## Abstract

Seed endophytes are of interest because they are believed to affect seed quality, and ultimately, plant growth and fitness. A comprehensive understanding of the assembly of the seed microbiome during seed development and maturation, the fate of microbes during storage, and the migration of microbes during seedling growth are still lacking. In this study, to understand the assembly and fate of endogenous bacteria in rice seeds from the ripening stage to the storage and seedling stages, we employed culture-dependent and metagenomic analyses. Bacterial communities in rice seeds were composed of a few dominant taxa that were introduced at the milky and dough stages, and they persisted during seed maturation. The culturable bacterial population gradually increased during the ripening stage, whereas there was a gradual decrease during storage. Bacteria that persisted during storage proliferated after imbibition and were distributed and established in the shoots and roots of rice seedlings. The storage temperature influenced the abundance of bacteria, which consequently changed the bacterial composition in the shoots and roots of seedlings. *Pantoea, Pseudomonas*, and *Allorhizobium* were consistently abundant from seed development to the germination stage. Some endogenous bacterial strains significantly promoted the growth of *Arabidopsis* and rice plants. Overall, our results indicate that rice seeds are colonized by a few bacterial taxa during seed development, and their relative abundance fluctuates during storage and contributes significantly to the establishment of endophytes in the stems and roots of rice plants. The selected bacterial isolates can be used to improve the growth and health of rice plants. To the best of our knowledge, this is the first study to reveal the dynamics of bacterial populations during storage of rice seeds at different temperatures. The temporal dynamics of the bacterial community during seed storage provide clues for the manipulation of endogenous bacteria in rice plants.

## Introduction

Plants host diverse microbial populations inside and outside of different compartments, such as vegetative organs (roots, stems, and leaves) and reproductive organs (flowers, seeds, and fruits) (Trivedi et al., 2020; Compant et al., 2021). The occurrence and abundance of microbial populations depend on the plant’s endogenous and environmental conditions provided by the different compartments, plant genotypes, and the surrounding environment (Compant et al., 2010; Hardoim et al., 2012; Truyens et al., 2015; Midha et al., 2016). It has been reported that endophytic strains that are competitive with adaptive colonization ability can inhabit reproductive plant organs, such as seeds (Okunishi et al., 2005; Truyens et al., 2015). The conditions within the seed change during seed development and maturation, permitting certain microbes to thrive and inhabit the seed (Herrera et al., 2016; L’Hoir and Duponnois, 2021). Certain seed-associated microbes colonize specific seedling compartments, while others colonize all tissues, representing a core microbiome (Johnston-Monje et al., 2021). The existence of a core seed microbiome suggests that certain bacterial endophytes adapt to physiochemical changes of seeds during maturation and storage (Johnston-Monje et al., 2021; Kim and Lee, 2021).

The dynamics of the seed microbiome during maturation, transferred to the next generation, and their influence on the seedlings have been the subject of many studies (Shade et al., 2017; Dos Santos et al., 2021; Kim and Lee, 2021; Bintarti et al., 2022). Endophytes of rice plants and seeds have also garnered recent attention. Kaga et al. (2009) recovered isolates closely related to *Bacillus firmus, Bacillus fusiformis, Bacillus pumilus, Caulobacter crescentus, Kocuria palustris, Micrococcus luteus, Methylobacterium fujisawaense, Methylobacterium radiotolerans*, and *Pantoea ananatis* from rice seeds, using a culture-dependent approach. A recent metagenomic study reported that *Pantoea*, *Methylobacterium*, *Curtobacterium*, *Pseudomonas*, and *Sphingomonas* are the dominant genera in rice seeds (Kim et al., 2020). The diversities of rice seed endophytes changes during maturation (Okunishi et al., 2005) and resilient endophytes are conserved, even when plants are grown in different geographical locations (Walitang et al., 2019). As seeds begin to germinate, seed endophytes may become important founders of the seedling microbial community. Rice seed germination changes the microbial community inherited from seeds and partitions it into the above- and below-ground tissues and the rhizosphere (Kim and Lee, 2021). After harvesting, rice seeds normally need to be dried artificially and stored in warehouse bags or silos for the next planting season. The seed endophytes that are likely to be inherited from the previous generation must survive the storage stage (Truyens et al., 2015). Despite many reports about microbiota in different growth stages and compartments of plants (Compant et al., 2011; Brader et al., 2014; Minervini et al., 2015; Torres- Cortés et al., 2018), the structure of rice seed microbiomes, temporal, and spatial dynamics of the bacterial populations during rice seed maturation, storage, and seedling development are not yet completely understood (Ali et al., 2021).

Both endophytic and epiphytic microbiota benefit plant growth and health by increasing the availability of mineral nutrients, producing phytohormones, and suppressing abiotic and biotic stresses (Bhattacharyya et al., 2015; Mohanram and Kumar, 2019; Jhuma et al., 2021; Mohanty et al., 2021). Seed endophytes have received particular attention as they may be important founders of the plant microbial community, which is essential for plant growth and health (Pitzschke, 2016; Shade et al., 2017; Eid et al., 2021). Midha et al. (2016) reported that *Curtobacterium, Enterobacter, Methylobacterium, Microbacterium*, and *Sphingomonas* that were isolated from rice seeds, promoted plant growth by supplying nutrients or phytohormones. Culturable rice seed endophytes like *Pseudomonas* strains, promote plant growth by nitrogen fixation, siderophore production, and phosphate solubilization (Walitang et al., 2017). Some endophytic bacteria, such as *Rhizobium, Pantoea, Sphingomonas*, and *Paenibacillus*, have been identified as core bacterial genera of rice seeds and have also been reported to promote growth and induce stress tolerance in rice plants (Mendis et al., 2018; Wang et al., 2020; Lu et al., 2021). More recently, endophytic *Sphingomonas melonis* in rice seeds was transmitted across generations to confer resistance against the seed-borne pathogen *Burkholderia plantarii* (Matsumoto et al., 2021).

Manipulation of plant-associated microbiomes has the potential to reduce chemical inputs, such as pesticides and fertilizers, resulting in more sustainable agricultural practices (Huang et al., 2016). Endophytic bacteria colonize plant reproductive organs like flowers, berries, and seeds (Compant et al., 2008, 2011), and the introduction of specific bacteria in flowers ensure bacterial colonization in progeny seeds (Mitter et al., 2017). These reports suggest that seed microbes could be used to design plant-beneficial microbial consortia; however, the potential for application of seed-borne endophytic bacteria remains underexplored (Eid et al., 2021; Orozco-Mosqueda and Santoyo, 2021). A thorough understanding of the dynamics of bacteria during seed storage and preparation of seeds for the next season’s cultivation would ensure the selection and maintenance of endogenous microbes that are beneficial for the growth and health of plants.

In this study, to understand the assembly and fate of bacterial populations in rice seeds, we employed culture-dependent and sequencing analyses to assess the temporal dynamics of bacterial communities during the ripening stage of rice seeds and storage at different temperatures. To track the distribution of endogenous bacteria from seeds to seedling compartments during germination and seedling development, we characterized the bacterial taxa in the shoot and root tissues of rice seedlings cultured under axenic conditions. Furthermore, we isolated and characterized potent bacterial strains with plant growth-promoting activities. To the best of our knowledge, this is the first study to reveal the dynamics of bacterial populations during storage of rice seeds at different temperatures.

## Materials and Methods

### Rice Cultivation and Environmental Conditions of the Paddy Fields

Rice (*Oryza sativa* L.) seeds of variety Shindongjin (SDJ) and Sukwang (SK) were sown in a nursery bed on May 20, 2020, and the seedlings were transplanted to the experimental fields at Jeonbuk Agricultural Research and Extension Services, Iksan, Jeollabuk-do, South Korea, on June 11, 2020. The altitude of the field was approximately 30 m with a mean temperature and rainfall of approximately 25°C and 225 mm, respectively, during the rice-growing season (June–August). Rice plants were cultivated based on Korean standard practices in paddy fields. The paddy field was irrigated with basal fertilizer incorporation during transplanting. Continuous flood irrigation took place during summer, and the field was drained approximately 20 days before the rice harvest. The plants received additional fertilizer in early June and mid-July. No pesticide was applied during cultivation, except for an herbicide treatment after transplanting.

### Sampling of Rice Panicles During the Ripening Stage and Assessment of Culturable Bacteria in the Seeds

**FIGURE 1:**
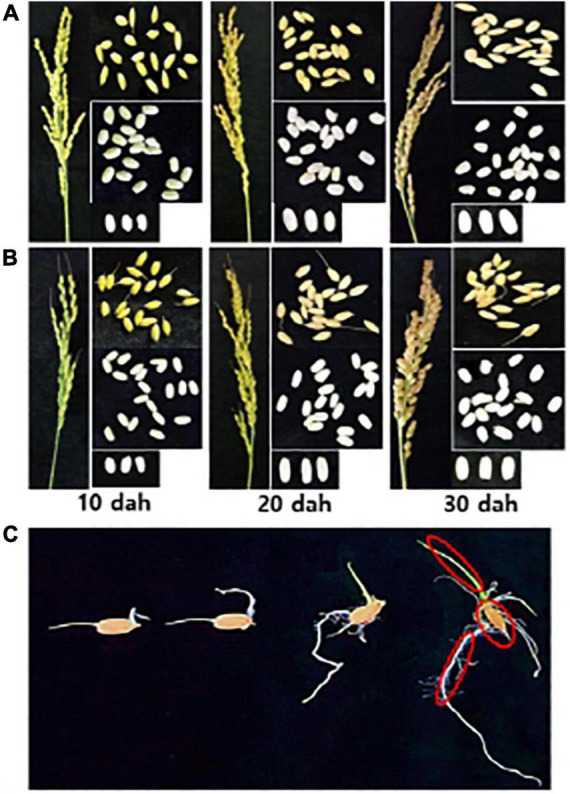
Sampling of rice seed and seedling compartments. Rice panicle and seeds of **(A)** Sukwang and **(B)** Shindonngjin were collected at three different stages of the ripening stage; 10 days after heading (dah; milky–dough ripening stage), 20 dah (dough–yellow ripening stage), 30 dah (yellow–full ripening stage). **(C)** Rice seeds were surface-sterilized and placed aseptically onto two layers of moist filter paper placed on culture plates. The seeds were incubated in a plant growth room under a 16 h light/8 h dark cycle at 28°C. Each compartment (circled in red), the seed remains, shoots (coleoptile), and roots (rhizoid) were collected 10 days after incubation. Bacteria and DNAs were isolated for the bacterial profiling from each sample.

Rice panicles were sampled from the paddy rice field at three different times during the ripening stage: 10 days after heading (dah; milky–dough ripening stage), 20 dah (dough–yellow ripening stage) and 30 dah (yellow–full ripening stage) (Figures 1A,B). Three panicles from three individual rice plants (spaced within 1 m) were collected and combined to form one biological replicate. The samples were placed in sterile plastic bags and stored at 4°C. The seeds were detached from the panicles after drying at room temperature for 2–3 days and were processed for the isolation of microbes. Half of the samples were frozen in liquid nitrogen and stored at −80°C until DNA extraction.

Endophytic bacteria were isolated from the seeds following the methods of Mano et al. (2006) and Hardoim et al. (2012) with minor modifications. Since rice grains were very soft during the first stage, the seeds (1 g; ca. 60 seeds) were surface-sterilized before the hulls were removed. This was performed by subjecting them to a solution (50 ml) containing 1% sodium hypochlorite (NaClO) for 5 min, washing them three times with sterile distilled water (sDW), and placing them peeled up in a laminar hood with sterilized forceps. From the second-stage samples, the seeds were surface-sterilized with 0.1% NaClO for 1 min, followed by rinsing with 70% ethanol. and two washes with sDW. The last wash was plated onto Luria-Bertani (LB) agar plates to check for contamination. The excess water from the sterilized rice seeds was removed using sterilized tissue paper, and the seeds were macerated in a sterilized mortar and pestle and suspended in saline solution (0.85% NaCl). The homogenates (10 g in 100 ml) were used for serial five-fold dilutions, which were plated onto 1/10 tryptic soy agar (TSA) with 1% rice seed exudates (RSE), prepared using the following procedure. For the preparation of RSE, rice grains harvested in the previous year were dehulled and surface-sterilized, as described above. The rice seeds were then kept in sDW (10% w/v) at 30°C under shaking conditions at 200 rpm for 4 h. The suspension was then passed through two layers of cheesecloth to remove the grains and filter-sterilized using 0.45 μm syringe filters. The RSE was plated on LB agar plates to confirm sterilization and was used for the preparation of bacterial isolation media. The plates with serially diluted suspensions (two duplicate plates for each dilution) were incubated at 30°C for 3 days, and the number of colonies was recorded. The bacterial colonies showing distinct morphotypes (colors and shapes) were randomly picked and transferred individually to a 1.5 ml tube containing LB medium with 15% (v/v) glycerol. The tubes were then incubated at 30°C for 24 h and stored at −80°C for further biological analysis. Some bacterial isolates that did not grow by direct incubation in LB broth were cultured using LB plates and diluted in LB medium with 15% (v/v) glycerol.

### Storage Conditions of Rice Seeds and Assessment of Culturable Bacteria During Storage

The seeds were detached from the panicles that were collected at 30 dah and air-dried for 5 days at room temperature. The seeds were placed in a thick paper envelope and stored at 4°C and 15°C. At 2, 4, and 6 months after storage (mas), the seeds were surface-sterilized with 0.1% NaOCl for 5 min, dehulled, and processed for the isolation of bacteria, as described above. The bacterial colonies showing distinct morphotypes were randomly picked and stored at −80°C. Half of the samples were frozen in liquid nitrogen and stored at −80°C until DNA extraction.

### Germination of Rice Seeds and Assessment of Culturable Bacteria From Each Compartment of Young Seedlings

Endogenous bacteria in the shoots, roots, and seed remains of young rice seedlings (Figure 1C) were isolated as follows. After 6 months of storage, the rice seeds were surface-disinfected and placed aseptically onto culture plates with two layers of sterilized moist filter paper. The rice seeds were incubated in a plant growth room under a 16 h light/8 h dark cycle (illumination at 3,000 Lux) at 28°C for 10 days, during which time shoots grew 8–10 cm with 2–3 leaves and roots grew 3–5 cm, leaving the seed remains. From each replicate, 40 seedlings were sampled and dissected into each compartment: seed remains (1 g), stem (coleoptile, 300 mg), and roots (500 mg). Each sample was surface-sterilized with 1% NaClO (seeds) or 0.1% NaClO (roots and stems) for 1 min, rinsed with 70% ethanol for 1 min, and washed with sDW for two times. To confirm that surface-sterilization was complete, the last wash was plated onto LB agar plates, and the plates were incubated at 28°C for 7 days, for which no bacterial growth was confirmed. Each compartment was macerated in a sterilized mortar and pestle. The homogenates were serial ten-fold dilutions and plated onto 1/10 TSA plates with 1% RSE. The plates were incubated at 28°C, and the colony number was assessed. Colonies showing distinct morphotypes were picked and stored at −80°C. Half of the samples were frozen in liquid nitrogen and stored at −80°C until DNA extraction.

### DNA Isolation and 16S rDNA Sequencing

The DNA was extracted from samples collected at each replicate using the GeneAll Exgene™ Soil DNA isolation kit (GeneAll, South Korea), following the manufacturer’s instructions. Briefly, to isolate DNA, rice seeds, shoots, and roots were surface-sterilized, as described above. The disinfected samples were crushed in a sterilized mortar and pestle before being placed in the PowerBead tubes of the kit. The isolated DNA was analyzed for purity using Bio-Tek, Epoch™ Spectrometer (United States), and DNA conditions were assessed by 1% agarose gel electrophoresis. To generate bacterial libraries, the PCR was performed to amplify the V3–V4 region of the 16S rRNA gene using fusion primers 341F (5′-AATGATACGGCGACCACCGA GATCTACAC-XXXXXXXXTCGTCGGCAGCGTC-AGATGTG TATAAGAGACAG-CCTACGGGNGGCWGCAG-3′) and 805R (5′-CAAGCAGAAGACGGCATACGAGAT-XXXXXXXXGTCT CGTGGGCTCGG-AGATGTGTATAAGAGACAG-GACTACH VGGGTATCTAATCC-3′), consisting of P5 (P7) graft binding and i5 (i7) index, NextEra consensus and adaptor sequences, and the target region sequence (Fadrosh et al., 2014). The PCR conditions were as follows: initial denaturation at 95°C for 3 min, followed by 25 cycles of denaturation at 95°C for 30 s, annealing at 55°C for 30 s, extension at 72°C for 30 s, and final extension at 72°C for 5 min. The PCR products were purified, and equal concentrations were pooled to remove short non-target fragments. The product quality and size were checked using an Agilent Bioanalyzer 2100 system. Purified amplicon libraries were pooled and sequenced at ChunLab, Inc. (Seoul, South Korea) with an Illumina MiSeq system using the MiSeq Reagent Kit v2 (Illumina Inc., United States). Sequence data are available in the GenBank SRA database under the BioProject accession number PRJNA000000.

### Sequence Data Processing and Analysis

The raw sequencing reads were filtered out for low quality (average quality value <25) reads using Trimmomatic v0.32. The paired-end sequences were merged using the fastq_mergepairs command of VSEARCH version 2.13.4 (Rognes et al., 2016), and primers were trimmed at a similarity cut-off of 0.8 (Myers and Miller, 1988). Unique reads were extracted, and redundant reads were clustered using VSEARCH. Taxonomic assignment was implemented by searching the EzBioCloud database (Yoon et al., 2017) using the VSEARCH program, and sequence similarity was calculated *via* pairwise alignment. The chimeric sequences were filtered on reads with <97% similarity by reference-based chimeric detection, using the UCHIME algorithm (Edgar et al., 2011), as well as the non-chimeric 16S rRNA database from EzBioCloud. Query sequences that matched the reference sequence at 3% sequence dissimilarity in EzBioCloud were identified at the species level. The species identified in the EzBioCloud database and OTUs obtained by the cluster database at high identity with tolerance (CD-HIT) and UCLUST were combined to form the final set of OTUs (Li and Godzik, 2006). Alpha diversity analysis (Chao1, Shannon, and phylogenetic diversity indices) and rarefaction curves were calculated using EzBioCloud, while beta diversity distances and principal coordinate analysis (PCoA) were analyzed by Jensen–Shannon algorithm (Lin, 1991). Differences in alpha diversity, including the number of operational taxonomic units (OTUs), richness, and diversity were analyzed, and the taxonomic composition was compared at the phylum-to-species level. The sequencing data for seeds at the ripening stage (20 dah), shoots, roots, and the seed remains of young seedlings were sufficient to cover detectable species, whereas additional sequencing was needed for stored seeds (4 mas) to sufficiently account for the diversity. Hence, we directly compared the bacterial diversity at the genus and species levels between samples at 20 dah and young seedlings, and the changes in specific genera and species were tracked and compared with samples at 4 mas. The relative differences in the bacterial populations were determined using the least significant difference (LSD) test at *P* = 0.05.

### *Arabidopsis* Growth Promotion Assay and Identification of Bacteria

The bacterial strains stored at −80°C were revived and cultured in LB broth at 28°C for 24 h. Bacterial cells were harvested by centrifugation and suspended in sDW amended with 0.2% sterilized carboxymethyl cellulose (CMC). *A. thaliana* ecotype Columbia-0 (Col-0) seeds were surface-sterilized with 70% (v/v) ethanol for 90 s and 1% (v/v) sodium hypochlorite for 5 min, then washed three times with sterile DW. Disinfected seeds were bacterized by soaking each bacterial suspension for 30 min. The seeds were placed in Petri dishes with sterilized filter paper to remove excess moisture. Seeds soaked in sDW amended with 0.2% CMC served as controls. After bacterization, *A. thaliana* seeds (5 seeds/plate) were sewn onto Petri dishes (90 × 15 mm) containing half-strength Murashige and Skoog (1/2 MS) medium, supplemented with 1.5% sucrose, and 0.8% (w/v) agar. The plates were sealed with parafilm and placed at an angle of 70° in plant-growth chambers under light-cycle (16-h light/8-h dark; 100 μmol m^–2^ s^–1^) conditions at 23 ± 1°C. After 10 days of incubation, the length of the root and the number of lateral roots of individual seedlings were measured. In addition, the fresh weight of plants (5 plants per replicate) was recorded. The experiment consisted of three replicates of five seeds each, and the entire experiment was repeated three times. To identify the bacterial strains to be selected for growth promotion in *Arabidopsis*, genomic DNA was isolated and sequenced with primers 27F and 1492R. The 16S rDNA sequences were aligned with sequences available in GenBank using the BLAST program.

### Growth Promotion Assay Using Rice Plants

Rice seeds were surface-sterilized with 2% sodium hypochlorite for 2 min, followed by five washes with sDW. The bacterial isolates selected from the *Arabidopsis* growth promotion assay were studied further to promote the growth of rice plants. Overnight-grown bacterial cells were collected and suspended (1 × 10^8^ CFU/ml) in sDW amended with 0.2% sterilized CMC. Surface-sterilized rice (cv. Shindonngjin) seeds were placed in a bacterial suspension for 1 h, followed by drying on a clean bench for 15 min. Seeds treated with 0.2% CMC in sDW served as controls. The treated seeds were sown in the soil in culture plates and cultivated at 27°C with 16 h light and an 8 h dark photoperiod for 2 weeks. The effect on growth promotion was recorded in terms of shoot and root length and plant weight.

### Plant Growth-Promoting Trait Assays

The selected bacterial strains were assayed for their growth-promoting activities. Siderophore production was assayed according to the modified chrome azurol S agar method (Schwyn and Neilands, 1987) by culturing the bacteria in LB media supplemented with CAS-FeCl3-HDTMA mixture and observing an orange halo in the media after incubation at 28°C for 4 days. Phosphate-solubilization ability was determined by inoculation in Pikovskaya’s agar medium by observing the clear zones around colonies after 3 days of incubation at 28°C (Pikovskaya, 1948). Silicate-solubilization ability was determined by observing a clear zone in the glucose medium (Lee et al., 2019). Indole acetic acid (IAA) production was assessed by growing the bacteria in a nutrient medium consisting of peptone, yeast extract, tryptone, and l-tryptophan, and the culture supernatant was mixed with Salkowski reagent to observe the color change (Apine and Jadhav, 2011).

### Statistical Analysis

The data for growth promotion analysis and estimation of culturable bacteria were subjected to analysis of variance using SAS JMP software (SAS Institute, Cary, NC, United States). Significant differences in the treatment means of each sample were determined using the LSD test at *P* = 0.05. Data from each experiment were analyzed separately.

## Results

### Changes of Culturable Bacteria in Rice Seeds During Ripening and Storage

**FIGURE 2:**
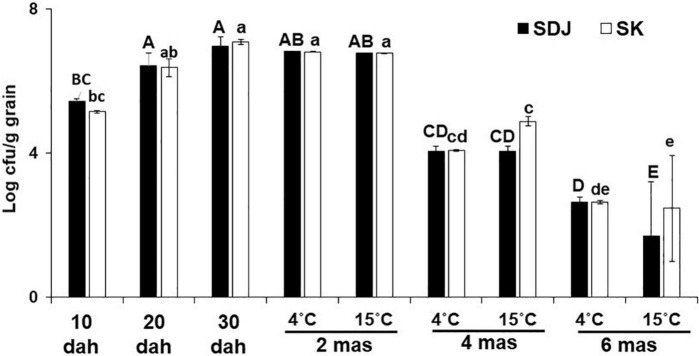
Total culturable bacterial populations in rice seeds during ripening and storage stages. Rice seeds of varieties Shindongjin (SDJ) and Sukwang (SK) were collected during the ripening stage, 10 days after heading (dah), 20 dah, and 30 dah; during storage, 2 months after storage (mas), 4 mas, and 6 mas from harvesting. Culturable bacteria were estimated in 1/10 TSA media amended with 1% RSE. Data represent mean ± SD and bars with the same letter do not differ significantly at *P* = 0.05 for each variety.

The population of endogenous microbiomes in the seeds of many crops changes during the development and maturation of the seeds. In this study, to understand the changes in the bacterial populations in rice seeds during the ripening stage and storage (Figure 1), the number of culturable bacteria was assessed using 1/10 TSA media supplemented with 1% RSE. The total culturable bacteria during maturation of the seeds of both rice varieties SDJ and SK gradually increased until 30 dah, with similar patterns of an approximately 1.2-fold increase every 10 days (Figure 2). During storage at 4 and 15°C, the culturable bacterial population was constantly maintained up to 2 mas at 30 dah without any significant fluctuations, irrespective of storage temperature (Figure 2). The number of culturable bacteria in the seeds was significantly decreased at 4 mas (1.7- and 1.7-fold decrease in SDJ and 1.7- and 1.5-fold decrease in SK at 4 and 15°C storage, respectively) and 6 mas (2.6- and 4.1-fold decrease in SDJ and 2.6- and 4.2-fold decrease in SK at 4 and 15°C storage, respectively) compared to 30 dah. The decrease in culturable bacteria at 6 months was significantly higher when the rice seeds were stored at 15°C (2.4- and 2.9-fold decrease at SDJ and SK, respectively, compared to 4 mas) than at 4°C (1.5- and 1.5-fold decrease at SDJ and SK, respectively, compared to 4 mas) (Figure 2). During seed formation, nutrients from the maternal seed coat are loaded into the growing endosperm (Martínez-Ballesta et al., 2020), which might influence the quantity and quality of the bacterial population during rice seed maturation. The decrease in culturable bacteria during storage might be due to the limited water content in the seeds with the passage of storage time. Overall, the results indicate that the culturable bacterial population increased during rice-seed ripening but significantly decreased during storage.

### Dynamics of Culturable Bacteria in Shoots, Roots, and Seed Remains After Germination

**FIGURE 3:**
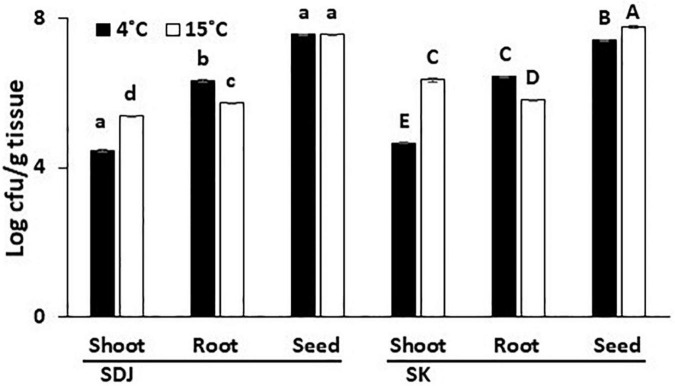
Total culturable bacteria in shoots, roots, and seed remains of rice plant seedlings. The rice seeds, Shindongjin (SDJ) and Sukwang (SK), stored for 6 months at 4 and 15°C were surface-sterilized and germinated on the moist filter paper. After 10 days of germination, bacteria were isolated in 1/10 TSA media amended with 1% RSE. Data represent mean ± SD and bars with the same letter do not differ significantly at *P* = 0.05 for each variety.

During the process of seed germination, the bacterial community in the seeds might migrate into the stems and roots of the seedlings and proliferate in specific tissues. To study the specific dispersion of bacteria during germination, we assessed the endogenous bacterial populations from the remains of the seeds, shoots, and roots of the rice seedlings that were aseptically cultivated from 6 months old seeds. The total culturable bacteria were higher in the seed remains than in the shoots and roots of the seedlings in both varieties (SK and SDJ), irrespective of storage temperature (4 and 15°C) (Figure 3). The total population in seed remains of SDJ seedlings obtained from seeds stored at 4 and 15°C were 3.9 × 10^7^ CFU/g and 3.8 × 10^7^ CFU/g, respectively, while seed remains of SK from 4 and 15°C stored seeds were 2.7 × 10^7^ CFU/g and 6 × 10^7^ CFU/g, respectively. The culturable bacterial numbers in the roots of SDJ and SK were higher than those in the shoots of seedlings. The culturable bacterial population in both varieties was maintained lower in shoots of plants grown from seeds stored at 4°C than at 15°C (2.8 × 10^4^ CFU/g and 2.5 × 10^5^ CFU/g for SDJ, and 4.6 × 10^4^ CFU/g and 2.4 × 10^6^ CFU/g for SK, respectively), whereas the numbers were higher in roots obtained from seeds stored at 4°C (2.2 × 10^6^ CFU/g and 2.7 × 10^6^CFU/g for SDJ and SK, respectively) than at 15 C (5.4 × 10^5^ CFU/g and 6.7 × 10^5^CFU/g, respectively) (Figure 3). The results indicated that the bacterial population in the germinating seeds was significantly increased as the nutrients were hydrolyzed and the bacteria partitioned differently into shoots and roots of seedlings. In addition, the storage temperature influences the bacterial composition of the seeds, which consequently changes the bacterial population in the shoots and roots of seedlings.

### Changes of Metagenomic Bacterial Profiles in Rice Seeds During Ripening and Storage

**FIGURE 4:**
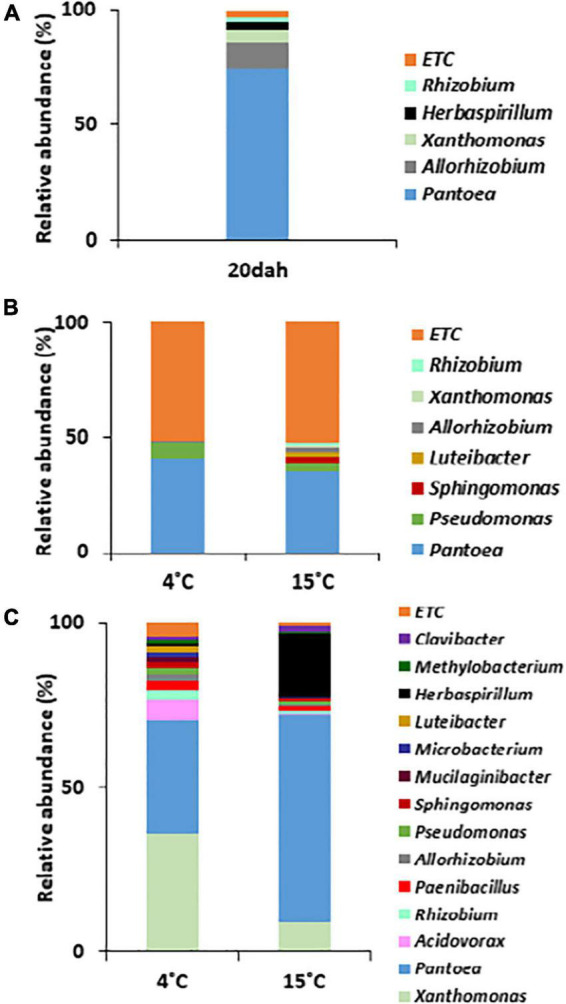
Comparison of bacterial composition in rice seeds at the genus level. The bacterial species with relative abundance >1% were compared between rice seeds of Shindongjin that are sampled **(A)** at 20 days after heading (dah), **(B)** 4 months after storage (mas) at 4 and 15°C, and **(C)** 10 days after germinated seeds stored for 6 months at 4 and 15°C.

To understand changes in bacterial community structures during the maturation and storage of rice seeds, we compared bacterial populations by metagenomic approaches between the rice seeds of SDJ sampled at 20 dah and 4 mas, and the seed remains after the germination of the seeds stored for 6 months. Among the bacterial population with relative abundance >1% at the genus level, the genus *Pantoea* was significantly dominant in rice seeds from the ripening stage (representing 74.8% of the reads) to storage (40.8 and 35.2% of seeds stored at 4 and 15°C, respectively, for 2 months) and the germination stage (34.2 and 62.7% in seed remains of seeds stored at 4 and 15°C, respectively, for 6 months), irrespective of the storage temperature (Figures 4A–C). Along with *Pantoea*, the genera *Pseudomonas* and *Allorhizobium* were consistently detected in all stages. The genera *Xanthomonas* (3.2%) and *Herbaspirillum* (1%) were abundant at the ripening stage (20 dah) but decreased to less than 1% relative abundance during storage. However, the population of both bacteria was significantly high in the germinated seeds that were stored at 4°C (36.2 and 1.3% for *Xanthomonas and Herbaspirillum*, respectively) and 15°C (9.3 and 19.4%, respectively) (Figure 4). Bacterial genera showing more than 1% relative abundance were significantly influenced by the storage temperature (Figure 4B), and eventually, the relative abundance of dominant bacterial populations was diversified during the germination stage (Figure 4C). *Pantoea* dominated with a relative abundance of 73.8% during the ripening stage and the dominance was similarly recorded for seedlings raised from seeds stored at 15°C (Figure 4C). However, *Xanthomonas* (3.1% during ripening) was the most dominant genus in seedlings (36.2%) grown from seeds stored at 4°C followed by *Pantoea* (34.2%). *Pseudomonas* and *Allorhizobium* (7.5% and 5.6%, respectively, during ripening) decreased in seedlings. Intriguingly, all bacterial genera showing >1% relative abundance in the seeds of the ripening stage were detected in seed remains after germination (Figures 4A,C).

**FIGURE 5:**
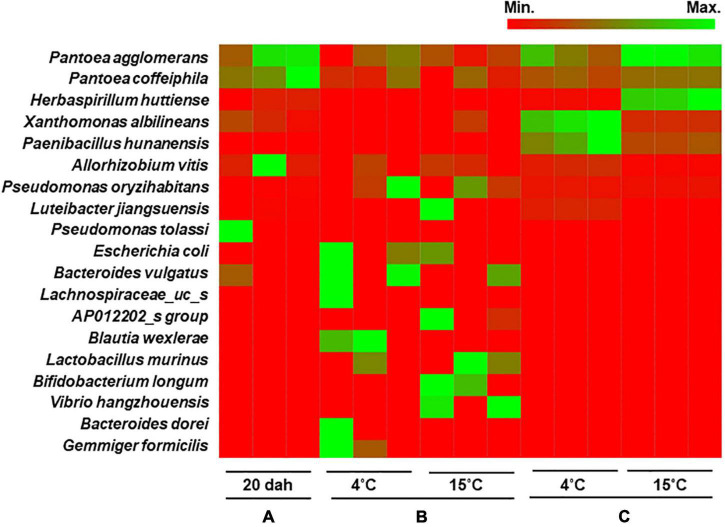
Heat map analysis of bacterial composition in rice seeds at species level. The bacterial community structure of the variety Shindongjin was compared between rice seeds that are sampled **(A)**, at 20 days after heading (dah); **(B)**, 4 months after storage (mas) at 4 and 15°C; and **(C)**, seed remains after 10 days of germination of 6-month-old seeds.

Heat map analysis showed that the bacterial composition at the species level was similar between seeds at the ripening stage and the seed remains in the rice seedlings (Figure 5). The bacterial population was dominated by *Pantoea agglomerans* with 55.5% relative abundance in seeds at 20 dah and 28.5 and 52.9% in the seed remains of seedlings grown from seeds stored at 4 and 15°C, respectively. During the storage (4 mas) of rice seeds, *Pantoea* species were the most abundant (>35% relative abundance irrespective of temperature), followed by *Allorhizobium vitis* and other bacteria, such as *Pseudomonas oryzihabitans* (Figure 5). *Xanthomonas albilineans* and *Paenibacillus hunanensis* were significantly higher in seed remains of seedlings cultivated from seeds stored at 4°C, whereas *Herbaspirillum huttiense* was the dominant species in germinated seeds stored at 15°C. Our results indicate that seed bacterial communities were composed of a few dominant taxa inhabiting the milky and dough stages, and they persisted during maturation. The assembled bacterial community structures changed during the storage and germination stages and were significantly influenced by the storage temperature of the rice seeds.

### Dynamics of Bacterial Taxa in Shoots and Roots of Rice Seedlings

Endogenous seed microbiota is the primary colonizer in the roots and shoots of plants. To understand the dispersion of bacterial populations from seeds to shoots and roots of rice seedlings and their proliferation in specific organs, we cultivated rice seedlings using seeds that were stored for 6 months at 4 and 15°C and analyzed the bacterial taxonomic composition by sequencing analysis in the shoots, roots, and seed remains. The species richness measured by Chao1 (Supplementary Figure 1A) and phylogenetic diversity (Supplementary Figure 1C) were higher in the seed remains than in the roots and shoots of seedlings grown from seeds stored at 4°C. There was no significant difference in the richness and diversity of the bacterial composition between the compartments of seedlings grown from seeds stored at 15°C. The species evenness based on the Shannon diversity index was high in the shoots of seedlings grown form seeds stored at 4°C (Supplementary Figure 1B). Meanwhile, the evenness was high in the seed remains and roots of seedlings grown from seeds stored at 15°C.

The PCoA analysis also showed that the respective bacterial composition of roots, shoots, and seed remains of seedlings grown from seeds stored at 4°C were distinct from those of seedlings grown from seeds stored at 15°C (Supplementary Figure 2A). The unweighted pair-group method with arithmetic means (UPGMA) clustering by Jensen–Shannon divergence metrics showed a greater difference in bacterial population among the roots, shoots, and seed remains in seedlings grown from seeds at 4°C compared to seedlings obtained from seeds stored at 15°C (Supplementary Figures 2B,C). The results indicate that the storage temperature significantly influenced the bacterial community structure and consequently influenced the bacterial structure in the specific organs of the seedlings.

**FIGURE 6:**
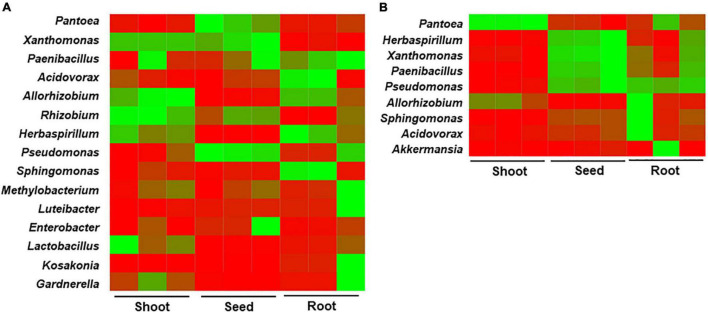
Heat map analysis for the bacterial community structure in compartments of rice seedlings. The rice seeds were stored at **(A)** 4°C and **(B)** 15°C for 6 months, germinated and cultivated for 10 days. Bacterial compositions were compared between the roots, shoots, and seed remains.

In seedlings obtained from seeds stored at 4°C, *Allorhizobium*, *Herbaspirillum*, and *Methylobacterium* were abundant in both roots and shoots. The genera *Paenibacillus*, *Acidovorax*, and *Sphingomonas* were abundant in roots, whereas *Xanthomonas*, *Rhizobium*, *Lactobacillus*, and *Gardnerella* were higher in shoots (Figure 6A), indicating the movement and proliferation of the bacteria in the respective organs. *Pantoea* and *Pseudomonas* were relatively abundant in the seed remains. In the case of seedlings obtained from seeds stored at 15°C, *Pantoea* was highly abundant in the shoots and roots (90.87 and 72.05% relative abundance, respectively) (Figure 6). *Sphingomonas* and *Acidovorax* were higher in the roots, whereas *Herbaspirillum*, *Xanthomonas*, *Paenibacillus*, and *Pseudomonas* were abundant in the seed remains, followed by the roots. The results indicate that differences in bacterial composition in rice seeds by storage temperature influenced the migration and enrichment of bacteria in the roots or shoots of rice seedlings.

### Selection of Potential Bacterial Strains That Promote the Growth of *Arabidopsis*

**FIGURE 7:**
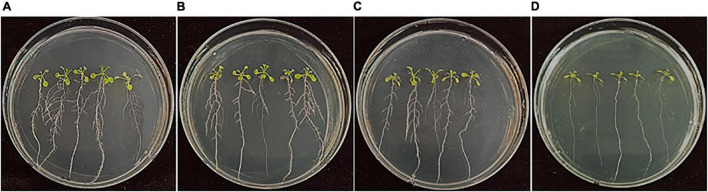
Growth promotion of *Arabidopsis* by treatment of bacterial strains isolated from rice seeds. *Arabidopsis thaliana* Col-0 seeds were surface-sterilized and bacterized by soaking in the bacterial suspension of **(A)** *Bacillus oceanisediminis*, **(B)** *Methylobacterium aquaticum*, and **(C)** *Williamsia muralis*. **(D)** The seeds treated with sterile DW amended with 0.2% CMC served as control. The bacterized seeds were sown onto 1/2 MS medium and cultured in plant-growth chambers. The photos were taken 10 days after incubation.

Many endogenous bacterial strains in rice seeds and the rhizosphere have been reported to promote the growth of rice plants. In this study, 229 bacterial isolates that were isolated from rice seeds were assessed for the growth promotion of *A. thaliana* Col-0. Among them, *Bacillus firmus, Bacillus oceanisediminis*, *Cytobacillus firmus*, *Massilia suwonensis*, *Methylobacterium aquaticum*, and *Williamsia muralis* enhanced shoot and root growth with increased lateral root numbers, compared to the untreated control (Figure 7). Among them, *B. oceanisediminis* isolated at the ripening stage and *W. muralis* and *M. aquaticum* isolated at 4 months of storage showed good performance in the increase in lateral roots and fresh weight (Supplementary Figure 3).

To understand the mechanisms underlying the growth promotion of *Arabidopsis*, we investigated bacterial strains for plant growth-promoting traits. The strains *B. firmus, B. oceanisediminis, C. firmus*, and *M. suwonensis* produced IAA and siderophores (Supplementary Figure 4), but no phosphate nor silicate mobilization activities. Overall, the results indicate that some endogenous bacteria are beneficial for the growth of plants with diverse traits.

### Growth Promotion of Rice Plants

**FIGURE 8:**
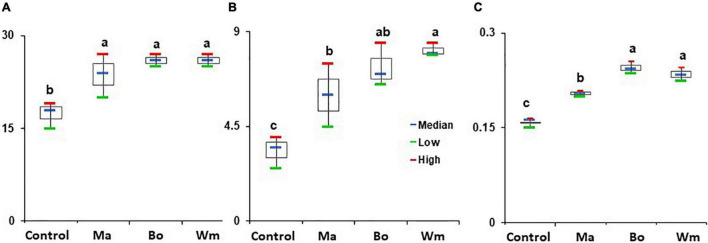
Effect of bacterial isolates on the growth of rice plants. Surface-sterilized rice seeds (cv Shindongjin) were soaked in 1 × 10^8^ CFU/ml of bacterial suspensions *Bacillus oceanisediminis* (Ba), *Methylobacterium aquaticum* (Ma), and *Williamsia muralis* (Wm). The seeds treated with sterile DW amended with 0.2% CMC served as control. **(A)** Shoot height (cm), **(B)** root length (cm), and **(C)** fresh weight (g) of rice plants were measured after 2 weeks of growth. The experiment consisted of three replicates of 10 seeds each, and the experiment was repeated three times. Red and green represent the high and low, respectively, whereas the horizontal blue bars within boxes represent the median. The tops and bottoms of boxes represent the 75th and 25th quartiles, respectively. Different letters indicate statistically significant differences among treatments.

The strains that promoted the growth of *Arabidopsis* were applied to rice seeds and assayed for the growth of rice plants. *B. oceanisediminis*, *M. aquaticum*, and *W*. *muralis* significantly increased the shoot height, root length, and fresh weight of rice plants compared to the untreated control (Figure 8 and Supplementary Figure 5). The growth patterns differed according to the treated strains. Both *B. oceanisediminis* and *W. muralis* increased shoot height (52.3% each), followed by *M. aquaticum* (41.9%), compared to the control. Root length was increased by treatment with *W. muralis*, *B. oceanisediminis*, and *M. aquaticum* by 147.9, 124.2, and 87.9%, respectively. Overall, the fresh weight increased by treatment with *B. oceanisediminis*, *W. muralis*, and *M. aquaticum* by 54.9, 48.9, and 30.9%, respectively, compared to the untreated control.

## Discussion

Plants are holobionts colonized with various microorganisms, including bacteria, fungi, archaea, and viruses. The microbiota in seeds play a crucial role in plant development and health. The complex interaction, diversity, richness, and dynamics of seed microbiome throughout the life cycle of plants are of substantial importance and require detailed analysis (Nelson, 2017; Berg and Raaijmakers, 2018; Santos and Olivares, 2021). However, our knowledge of the temporal and spatial dynamics of bacterial populations from seed development to the seedling stage is still lacking, and the dynamics of microbes during rice seed storage, which is important for the preparation of healthy seeds for the next planting season, has been underexplored.

Several changes occur during rice seed maturation, including nutritional alterations and water loss, resulting in a dry and quiescent seed with a starch-rich endosperm and an embryo headed for dormancy (An et al., 2020). The specific microhabitats associated with the seed and loss of water during the seed maturation process might also favor endophytes that are tolerant to high osmotic pressure (Mano et al., 2006; Chesneau et al., 2020). Each compartment of rice seeds possesses distinct microbial compositions, suggesting that seeds can differentially transmit microbiomes to different plant compartments (Mano et al., 2006; Eyre et al., 2019). Taken together, the physiochemical alterations during rice seed maturation influence the structure of plant endophytes. In this study, to track the assembly and fate of rice seed-borne bacteria during maturation and storage, we first assessed culturable bacteria. The total culturable bacteria in rice seeds gradually increased from 10 to 30 dah in the ripening stage, and the bacterial population size was maintained up to 2 mas without any significant fluctuations at either 4 or 15°C. Okunishi et al. (2005) reported that the diversity of rice seed endophytes decreases from early to late maturation stages. Recently, Eyre et al. (2019) indicated that bacterial diversity was low during seed development in rice plants. In addition, limited phylogenetic diversity was observed within seed bacterial communities of bean (*Phaseolus vulgaris*) and radish (*Raphanus sativus*) during seed filling and maturation, whereas phylogenetic diversity increased in the last stages of seed maturation for both bean and radish (Chesneau et al., 2020), which is consistent with the results of this study.

In many rice-growing countries, such as South Korea and China, rice seeds are dried artificially at high temperature after harvesting and stored for around 6 months for the next season. Low temperatures have been recommended for storage of seeds to avoid temperature-mediated changes in oxygen concentration, relative humidity, and enzymatic activity during storage, which might alter the physiochemical status of seeds, thereby, impacting seed longevity (Rajjou and Debeaujon, 2008; Wang et al., 2017, 2018). The changes in water, oxygen, and nutrient content of the stored seeds might also influence the residing endophytes. In recent reports, the bacterial communities in soybean seeds were altered by storage temperatures, and the genera with low abundance rapidly disappeared from seeds stored at room temperature and 4°C compared to seeds stored at −20°C (Chandel et al., 2021). In this study, the decrease in culturable bacteria at 6 mas was significantly higher when the rice seeds were stored at 15°C than when stored at 4°C. This might be due to the changes in water and nutrient conditions in the seeds as storage time increased. Overall, the results of our study indicate that the bacterial population increased during the development and maturation of rice seeds, was maintained for a couple of months of storage, and significantly decreased at 4 and 6 mas.

In previous reports, the culturable bacterial endophytic population estimates ranged from 10^1^ to 10^2^ CFU/g seed to as high as 10^6^–10^8^ CFU/g seed (Truyens et al., 2015; Nelson, 2017). No colonies appeared when surface-sterilized and homogenized *Passiflora edulis* (passion fruit) seeds were placed on the bacterial growing agar media because of the bacteriostatic activity of piceatannol. Meanwhile, colonies were generated from cut seedlings derived from *P. edulis* seeds (Ishida and Furuya, 2021), indicating that many seed bacteria remained uncultured. When we isolated bacteria using nutrient media (TSA) at a low concentration (1/10), very few colonies (<30) were observed. However, the colony numbers increased to as high as 10^5^–10^7^ CFU/g seed when media was supplemented with RSE, and the colonies appeared earlier and grew faster in 1/10 TSA media supplemented with RSE than in 1/10 TSA only. Most colonies from the seeds at 4 mas were embedded in the media and grew slowly, indicating changes in culturable bacteria and their physiology. Taken together, the results suggest that to reproducibly assess the microbial population in seeds, isolation procedures for endogenous bacteria in seeds need to be chemically and conditionally optimized to mimic the seed microhabitat.

The analysis of various plant species, such as sugarcane, grapes, and cactus, indicates that plant endophytic communities are frequently enriched in members of Proteobacteria and Firmicutes, and to a lesser extent, Bacteroidetes (Trivedi et al., 2020). In an earlier report, *Bacillus, Curtobacterium, Methylobacterium, Sphingomonas, Xanthomonas*, and *Micrococcus* were commonly present both inside and on the surface of rice seeds (Mano et al., 2006). Among them, three genera (*Paenibacillus, Acidovorax*, and *Pantoea*) and two genera (*Stenotrophomonas* and *Rhizobium*) were specific to the interior and surface of the seeds, respectively. The culturable bacteria isolated from rice seeds and identified by 16S rRNA gene sequencing were *B. firmus, B. fusiformis, B. pumilus, C. crescentus, K. palustris, M. luteus, M. fujisawaense, M. radiotolerans*, and *P. ananatis*. *Methylobacterium* and *Pantoea* have been frequently detected in both rice seedlings and mature rice plants (Kaga et al., 2009). In this study, *Pantoea* was significantly dominant, and *Pseudomonas* and *Allorhizobium* were consistently detected in rice seeds from maturation (20 dah) to storage (4 mas) and the germination stage (6 mas), irrespective of the storage temperature. In recent studies, *Pantoea* and *Pseudomonas* species were found to be abundant in rice seeds and seedlings (Kim and Lee, 2021). Strains of *Pantoea* and *Pseudomonas* belong to a core seed transmitted microbiome found in all angiosperm plants (Johnston-Monje et al., 2021), which explains why these strains were dominant in our samples. The genera *Xanthomonas* and *Herbaspirillum* were abundant at the ripening stage and in seed remains after germination, although the abundance was below 1% during storage. This result indicated the necessity to analyze the alteration of bacterial abundance over time with the emphasis on low abundance bacteria in seeds, which might also include genera that are important for germinating seedlings. In a recent study, Proteobacteria (83.8%: Gammaproteobacteria, 60.1%; Alphaproteobacteria, 12.5%) was the single prevalent phylum, and *Pantoea* (42.5%), *Methylobacterium* (11.8%), *Curtobacterium* (9.3%), *Pseudomonas* (8.7%), and *Sphingomonas* (8.6%) were the dominant genera in 26 domesticated and 17 wild rice collections worldwide (Kim et al., 2020). Overall, the results suggest that bacteria that are well-adapted to the ecological conditions of seeds can enter the seed interior, and the physiological changes that occur during seed development and maturation could increase the relative abundance of seed-endogenous bacterial species. Consequently, specific and distinct bacterial endophytes are conserved in the microhabitat.

Seed microbiota composition influences the structure of plant-associated microbial communities through the order and timing of species immigration during community assembly (Fukami, 2015). In rice seeds, the bacteria that colonize the seed interior are also important founders of the bacterial community present during the early plant growth stages (Hardoim et al., 2012), and become the dominant endophytic species in the mature plant and infect the subsequent generation *via* rice seeds (Kaga et al., 2009). In this study, we analyzed the contribution of rice seed-borne bacteria to the endophytic population in the shoots, roots, and remaining seeds of aseptically cultivated young seedlings. Our results showed that the bacterial population was higher in the remains, shoots, and roots of young seedlings than in stored seeds, suggesting that unculturable or undetectable bacteria may be sustained during storage and proliferate after water imbibition. The culturable bacterial population was lower in shoots and higher in the roots of seedlings grown from seeds stored at 4°C compared to seedlings grown from seeds stored at 15°C. Plant organs, such as seeds, roots, stems, and leaves, exhibit different chemical and ecological environments, which lead specific microorganisms to colonize the tissues (Compant et al., 2010). Wang et al. (2020) indicated that the tissue compartment is the strongest driving force shaping the microbial community in rice plants, as opposed to genotype, growth location, and harvest year. For instance, in rice plants, *Methylobacterium* from the shoots, *Burkholderia* and *Rhizobium* from the roots, *Azospirillum* and *Herbaspirillum* from the stems and roots, and *Pantoea* from the seeds have been commonly isolated (Mano and Morisaki, 2008). The composition of rice endophytic bacterial and fungal communities between above- and below-ground plant compartments is different due to differences in niches (Kim and Lee, 2021). Baldoni et al. (2016) suggested that metabolic differences between above- and below-ground compartments in rice plants might contribute to the differences in bacterial composition in each compartment. They also reported that seed bacteria moved to the leaves, stems, and roots; however, in field conditions, the root endophytic composition was influenced by soil microbiome (Baldoni et al., 2016). The results suggest that the storage temperature and culturing environment influence the bacterial population in the seeds, which consequently changes the population in the shoots and roots of seedlings. Rice seeds are dried at high temperature after harvesting and stored at room temperature through winter until the next sowing season. The alterations of the microbiome by storage temperature suggest that further studies need to be conducted to determine how storage conditions influence the rice seed microbiome and rice plant health under field conditions.

We further evaluated the seed-to-seedling transmission of seed-borne bacterial taxa. In the seedlings obtained from seeds stored at 4°C, *Allorhizobium, Herbaspirillum*, and *Methylobacterium* were abundant in both roots and shoots, whereas *Paenibacillus, Acidovorax*, and *Sphingomonas* were abundant in roots and *Xanthomonas, Rhizobium, Lactobacillus*, and *Gardnerella* were high in shoots. In the seedlings obtained from seeds stored at 15°C, *Pantoea* was highly abundant in shoots and roots, whereas *Sphingomona*s was high in roots. *P. agglomerans, Pantoea coffeiphila*, and *A. vitis* were abundant in the seeds from the ripening stage to the seedling stage. *X. albilineans* was abundant in all surveyed stages except in seeds stored at 4°C, and *P. hunanensis* was highly detected in all the compartments of seedlings obtained from seeds stored at both temperatures, excluding shoots of seedlings grown from seeds stored at 15°C. *H. huttiense*, which was abundant in the seeds at the ripening stage, was the dominant species in the seed remains of seedlings from seeds stored at 15°C, whereas it was more abundant in roots and shoots compared to seed remains of seedlings from 4°C stored seeds. *P. oryzihabitans* was detected at <1% relative abundance during the ripening stage and was dominant during storage. Overall, the results indicated that differences in bacterial composition in rice seeds by storage temperature could influence the enrichment and structure of bacteria in the roots or shoots of rice seedlings. Seed microbiota is acquired through horizontal and vertical pathways (Chesneau et al., 2020). The environment, such as the soil microbiome, is an important driver for the assembly and structure of the seed and seedling microbiota (Hardoim et al., 2012; Morales Moreira et al., 2021). In maize, the seed-to-seedling transmission was influenced by the microbiota of seed surfaces, which, in turn, affected germination speed and seedling growth (Dos Santos et al., 2021). In another study, soybean microbiome recovery after disruption was modulated by the seed microbiome and not the soil microbiome (Moroenyane et al., 2021). Seedling microbiomes of 17 different plant species, including rice, were dominated by seed-transmitted bacteria and some fungi (Johnston-Monje et al., 2021). Many studies have reported that plants maintain an intrinsic microbiome independent of soil type, host genotype, and environmental factors. The growth of plants under axenic conditions in this study supports the previous findings that the initial bacterial microbiota in the seeds largely determines the endophytic composition of the seedlings. In this study, *P. agglomerans, P. oryzihabitans*, and *A. vitis* were consistently conserved in rice seeds from the seed development to the seedling stage approximately 8 months after seed initiation. *X. albilineans* can also be considered as an intrinsic strain from the seed to the seedling stage, despite the lack of detection during storage at 4°C. Overall, our study indicates that storage temperature influences the migration of bacteria from seeds to shoots and roots and the consequent abundance in each compartment of germinated seedlings. The impact of changes in bacterial composition on the overall plant health and development needs further exploration.

Many endophytic microbes isolated from rice plants grown in various geographical locations and cultivation conditions have been reported to increase the production and health of rice plants (Cottyn et al., 2009; Wang et al., 2020). In this study, we screened strains that promoted the growth of *Arabidopsis*, and studied their plant growth-promoting traits. The strains selected from the *Arabidopsis* screening were further assayed using rice plants and identified by 16S rDNA sequencing. *B. firmus*, *B. oceanisediminis*, *C. firmus*, *M. suwonensis*, *M. aquaticum*, and *W. muralis* promoted the growth of *Arabidopsis*, and *B. oceanisediminis, M. aquaticum*, and *W. muralis* enhanced rice plant growth. *B. firmus*, which was developed as a commercial bioproduct, colonized plant roots, promoted plant growth, and protected against pathogens and nematodes (Mendis et al., 2018). *Methylobacterium aquaticum* has been reported to enhance the growth of rice, barley, and sugarcane (Rossetto et al., 2011; Tani et al., 2015). *B. oceanisediminis* contains *nif* genes for nitrogen fixation (Yousuf et al., 2017). The rice endophyte *Pantoea ananatis* D1 was reported to improve the salt tolerance of rice seedlings (Lu et al., 2021), and our study also identified *Pantoea* as the most abundant species. However, some of the *Pantoea* isolates tested for their effect on rice or *Arabidopsis* did not influence the growth significantly. *Williamsia* has been isolated from various parts of plants, including eucalyptus tree roots, as endophytes (Kaewkla and Franco, 2013); however, its interaction with plants and beneficial traits are not yet completely analyzed. To the best of our knowledge, *W. muralis* has not yet been studied as a plant growth-promoting agent.

Designing endophytic seed microbiota using beneficial microbes is a promising tool for improving plant growth and health. In this study, the assembly and temporal dynamics of bacterial populations in rice seeds were assessed from the seed development and storage stages to the young seedling stage. Culturable bacterial populations gradually increased during seed development and maturation. The number of endophytic bacteria gradually decreased with different fates at different storage temperatures. Although Chandel et al. (2021) reported the effect of storage temperature on seed microbiomes of soybean, to the best of our knowledge, this is the first study to track the dynamics of bacterial populations during rice seed storage. All bacterial genera that were dominant during the maturation of rice seeds were abundantly recovered in the seed remains after germination. Johnston-Monje et al. (2016) reported that the rhizosphere of plants grown in non-sterile soils had more bacterial diversity than that of plants grown in sterile soils, indicating the inclusion of soil bacteria in the rhizosphere. Kim and Lee (2021) reported dominant seed-borne OTUs in aboveground plant compartments, while roots were contributed by the soil microbial community under field conditions. In this study, the rice seedlings were grown under axenic conditions for 10 days, which eliminated the direct interference from soil microbes and interaction with soil microbiota, as well as changes in the seed microbiome with respect to the effect of seed and root exudates. Therefore, further studies under field conditions during the entire growth season are needed to understand the dynamics of the bacteria throughout the life cycle of rice.

The information obtained in this study may contribute to the manipulation of endogenous bacteria in the preparation of healthy seeds for the next planting season. Although little is known about the contribution of seed endophytes to the health and productivity of plants, the bacterial isolates may find use in the development of inoculants for sustainable agriculture in the future. The diverse factors that determine the assembly of seed microbiota and their fate require further study. The temporal and spatial dynamics of bacterial endophytes in mature plants cultivated from seeds stored under various environmental conditions require further investigation. We will study the application of selected strains for rice growth and health in the near future.

## Data Availability Statement

The name of the repository and accession numbers can be found below: NCBI; PRJNA816647, PRJNA816637, PRJNA816618, PRJNA816633, and PRJNA816614.

## Author Contributions

SD performed the experiments, analyzed the data, prepared the figures and tables, and drafted the manuscript. SC contributed to sampling and reviewed drafts of the manuscript. YL contributed to the design, analysis, interpretation of the data, and critically revised the manuscript. All authors approved the submitted version.

## Conflict of Interest

The authors declare that the research was conducted in the absence of any commercial or financial relationships that could be construed as a potential conflict of interest.

## Publisher’s Note

All claims expressed in this article are solely those of the authors and do not necessarily represent those of their affiliated organizations, or those of the publisher, the editors and the reviewers. Any product that may be evaluated in this article, or claim that may be made by its manufacturer, is not guaranteed or endorsed by the publisher.
