# Supplementary material for: Temporal Dynamics of Endogenous Bacterial Composition in Rice Seeds During Maturation and Storage, and Spatial Dynamics of the Bacteria During Seedling Growth
Source: Front Microbiol. 2022 Jul 22;13:877781. doi: 10.3389/fmicb.2022.877781 (PMC9355576; doi:10.3389/fmicb.2022.877781)
Supplement: Supplementary file 1 [file Presentation_1.pdf]

Supplementary Material

1 Supplementary Figures and Tables

1.1 Supplementary Figure

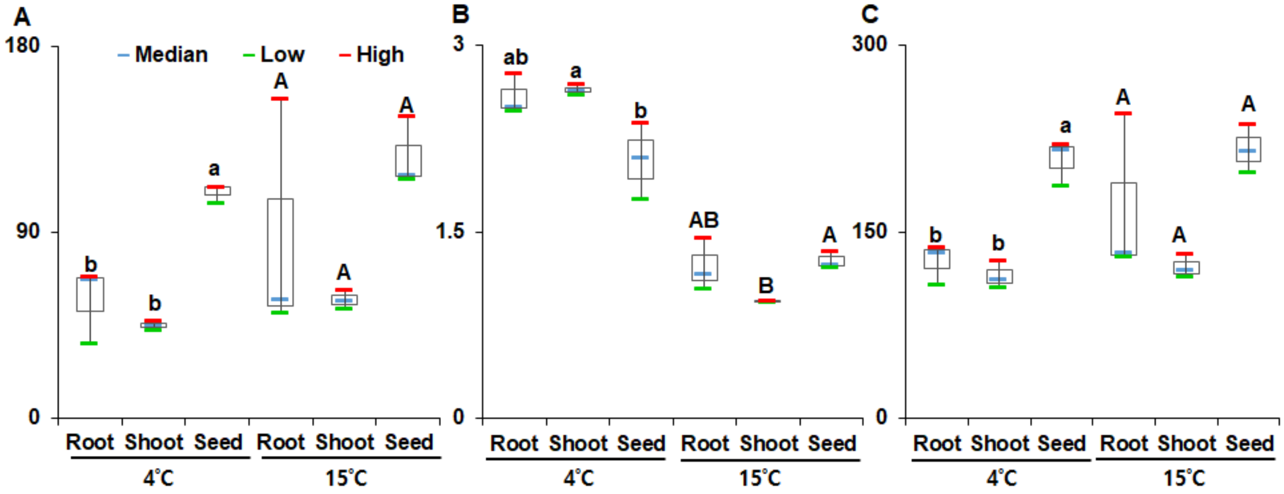

Figure S1. Comparison of richness and diversity of bacterial communities between compartments of seedlings grown from seeds stored in 4 °C and 15 °C. (A) Richness was estimated by Chao1, (B) species evenness was represented by Shannon's index, and (C) biodiversity incorporating phylogenetic difference between species was measured through phylogenetic diversity. Red and green represent the high and low, respectively, whereas the horizontal blue bars within boxes represent the median. The tops and bottoms of boxes represent the 75th and 25th quartiles, respectively. Different letters indicate significant difference between compartments of seedlings grown from seeds stored in a particular temperature at P = 0.05.

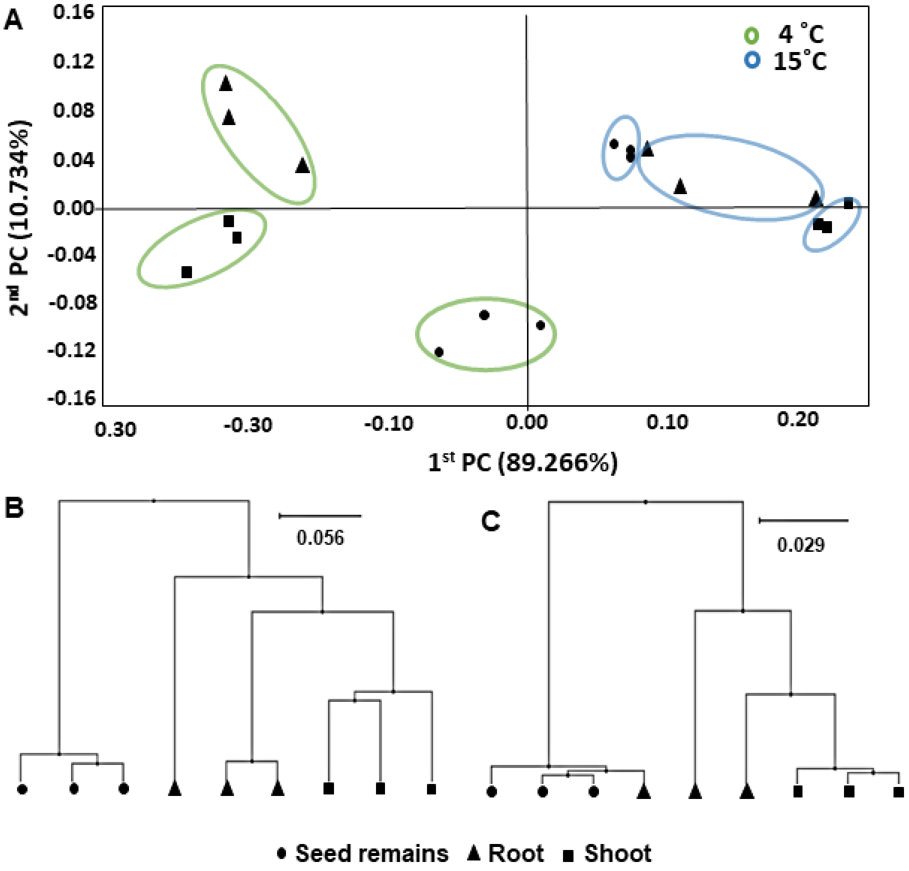

Figure S2. Principal coordinates analysis (PCoA) of bacterial community (A) and UPGMA clustering between seedling compartments. The rice seeds stored for 6 months at (B) 4 °C and (C) 15 °C were germinated and cultivated for 10 days in axenic conditions, and the bacterial community structure was compared between the roots, shoots, and seed remains by 16S rDNA sequencing. PCoA and UPGMA clusters were analyzed by Jensen–Shannon divergence metrics using relative abundances of all OTUs.

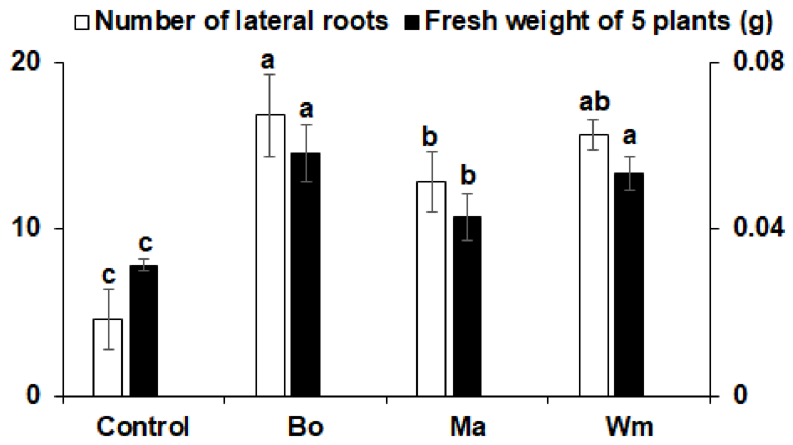

Figure S3. Growth promotion of *Arabidopsis* by treatment of the selected strains. The surface sterilized *Arabidopsis* seeds were treated with bacterial suspension of (A) *Bacillus oceanisediminis*, (B) *Methylobacterium aquaticum*, and (C) *Williamsia muralis*. The bacterized seeds were sown onto 1/2MS medium and cultured in plant-growth chambers. The seeds treated with sterile DW amended with 0.2% CMC served as control. Data represent mean ± SD and bars with same letter do not differ significantly at P = 0.05.

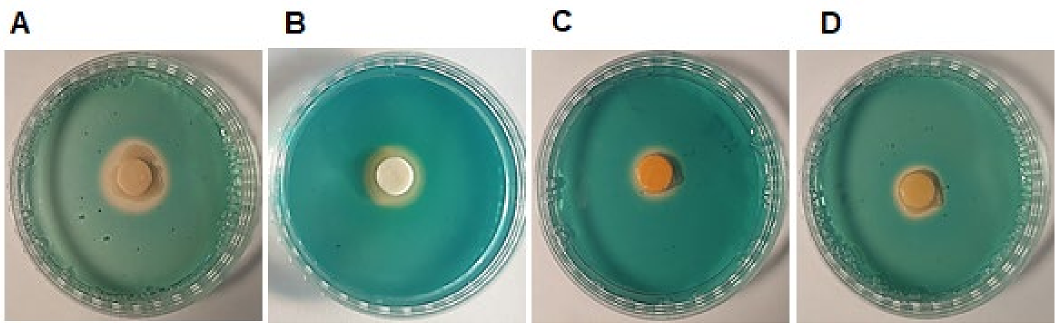

Figure S4. Siderophore production by selected bacterial strains. Overnight grown bacterial culture were spot inoculated with (A) *Cytobacillus firmus*, (B) *Bacillus firmus*, (C) *Bacillus oceanisediminis*, and (D) *Massilia suwonsis* onto the sterile paper discs laid on the CAS plates, and the plates were incubated at 30°C for 4 days. Siderophore production was assessed by a change in the color of CAS medium from blue to orange.

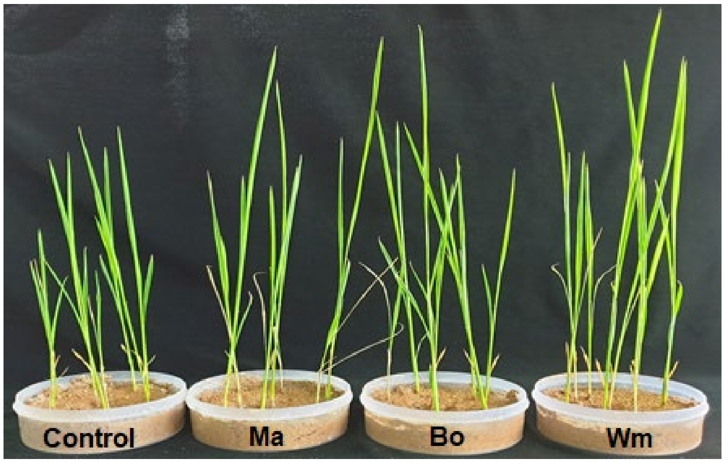

Figure S5. Effect of selected bacterial isolates on the growth of rice plant. Surface-sterilized rice seeds (cv Shindongjin) were soaked in 1×10^8 CFU/ml of bacterial suspensions *Methylobacterium aquaticum* (Ma), *Bacillus oceanisediminis* (Bo), and *Williamsia muralis* (Wm). The seeds treated with sterile DW amended with 0.2% CMC served as control.
